# Dramatic Response to Lorlatinib in a Patient With *CD74-ROS1*-Positive Lung Adenocarcinoma With Acquired F2004V Mutation

**DOI:** 10.1200/PO.19.00013

**Published:** 2019-06-03

**Authors:** Anastasios Dimou, Sai-Hong I. Ou, Robert C. Doebele

**Affiliations:** ^1^ University of Colorado School of Medicine, Aurora, CO; ^2^ University of California School of Medicine, Irvine, Orange, CA

## INTRODUCTION

A significant subset of patients with non–small-cell lung cancer (NSCLC) harbor activating mutations in key oncogenes like *EGFR*, *ALK*, *ROS1*, *NTRK*, *BRAF*, and others and account for up to one-quarter of the population with this common type of cancer.^1^ These oncogene-driven tumors are amenable to treatment with targeted therapies; however, secondary resistance invariably ensues. At the molecular level, resistance depends on activation of bypass pathways or development of secondary mutations in the driver oncogene.^2,3^

Oncogenic fusions involving the *ROS1* gene are detected in approximately 1% to 2% of patients with NSCLC and the majority of cases occur in tumors of adenocarcinoma histology.^4^ They are generated by the fusion of the 3′ end of the *ROS1* gene containing the tyrosine kinase with a variety of partner genes, including *GOPC*, *SLC34A2*, *CD74*, *TPM3*, *SDC4*, *EZR*, *LRIG3*, *KDELR2*, *CCDC6*, among others.^4^ ROS1 fusions function as primary oncogenic drivers following the oncogene addiction model.^4^ Indeed, inhibition of ROS1 with the small molecule tyrosine kinase inhibitor (TKI) crizotinib induced durable responses in 72% of the patients with an ROS1-positive NSCLC in the PROFILE-1001 study (ClinicalTrials.gov identifier: NCT00585195),^5^ leading to approval of crizotinib by regulatory agencies for this patient population. Other TKIs, including ceritinib,^6^ lorlatinib,^7^ and entrectinib,^8^ have shown activity in this patient population as the field evolves.

Our understanding of the landscape of resistance mechanisms for ROS1-positive NSCLC is still evolving. Although several TKI options currently exist for patients with ALK-positive NSCLC and secondary *ALK* mutations, effective TKIs are limited for the ROS1-positive lung cancer population in the postcrizotinib setting.^9,10^ Importantly, the molecular mechanisms of resistance are variable, which poses a barrier for the study of different drugs or drug combinations in molecularly defined cohorts of progressing patients in the form of clinical trials. Here, we describe an unusual case of a ROS1-positive patient who developed a *ROS1* F2004V secondary mutation and whose cancer progressed in the brain and bone sites after 34 months of treatment with entrectinib, followed by dramatic response to lorlatinib. Informed consent for use of the radiology images was obtained by the patient presented.

## CASE REPORT

A 22-year-old female patient with no prior medical history was diagnosed with metastatic lung adenocarcinoma after presenting with right-eye vision changes (flashing). An ophthalmologic examination revealed a right amelanotic perifoveal choroidal tumor. Subsequent evaluation with positron emission tomography/computed tomography scanning confirmed metastatic disease with multiple left lung nodules, left hilar lymphadenopathy, and bone lesions. Pathologic review of a core biopsy specimen from a lung lesion showed lung adenocarcinoma with predominant lepidic pattern. Molecular analysis with a commercial next-generation sequencing panel identified a *CD74-ROS1* fusion gene. Interestingly, choroidal metastases have been reported in a number of cases of ALK^11,12^ or other oncogene-driven lung cancer.^13^The patient was treated with radiation therapy to the metastatic lesion in the right eye, followed by first-line crizotinib. Although there was initially a complete response, progression of disease in the brain was documented while she was receiving crizotinib therapy for 10 months. She was then treated with entrectinib, a TKI that penetrates the blood-brain barrier, in the context of the phase I STARTRK-1 clinical trial (ClinicalTrials.gov identifier NCT02097810). The patient was initially treated at the recommended phase II dose of 600 mg orally once daily for the first eight 28-day cycles, but she experienced asymptomatic progression in multiple, small brain metastases, which led to a decision to escalate the dose to 800 mg orally once daily, resulting in decrease in disease burden in the brain. After another 18 cycles of entrectinib, an increase in the size of the brain lesions was noted. Progressing disease sites in the brain were treated with stereotactic ablative radiation, based on data that demonstrated extension of duration of benefit for TKIs in the setting of oligoprogression.^14^ The patient continued receiving entrectinib for a total of 34 months, until further progression in the brain developed with concomitant progression in osseous metastases.

At the time of brain and bone progression, a secondary *ROS1* F2004V mutation was detected via circulating cell-free DNA testing using a commercial next-generation sequencing assay. Besides the *CD74-ROS1* fusion and the F2004V secondary mutation, no other mutations were detected in the *ROS1* kinase domain or other genes. At that time, the patient was removed from the STARTRK-1 study and therapy was begun with brigatinib, a compound approved for the treatment of lung cancers with ALK fusions^15^ but also with activity against ROS1.^16,17^ In addition, brigatinib had activity against *CD74-ROS1* with F2004L in vitro.^18^ The patient also underwent radiation therapy for several thoracic spine progressing lesions. Unfortunately, follow-up scans showed disease progression in the brain and several osseous disease sites (outside of the radiated field).

**FIG 1.**
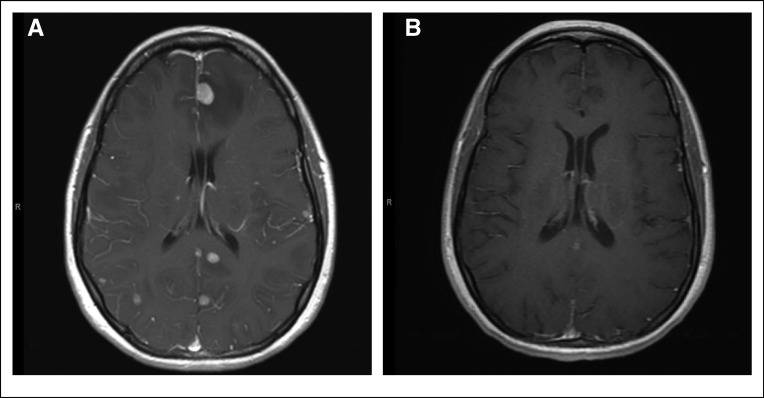
Magnetic resonance images of the brain (T1 axial postcontrast series) (A) before and (B) 1 month after treatment with lorlatinib, showing significant response of all metastatic brain lesions.

**FIG 2.**
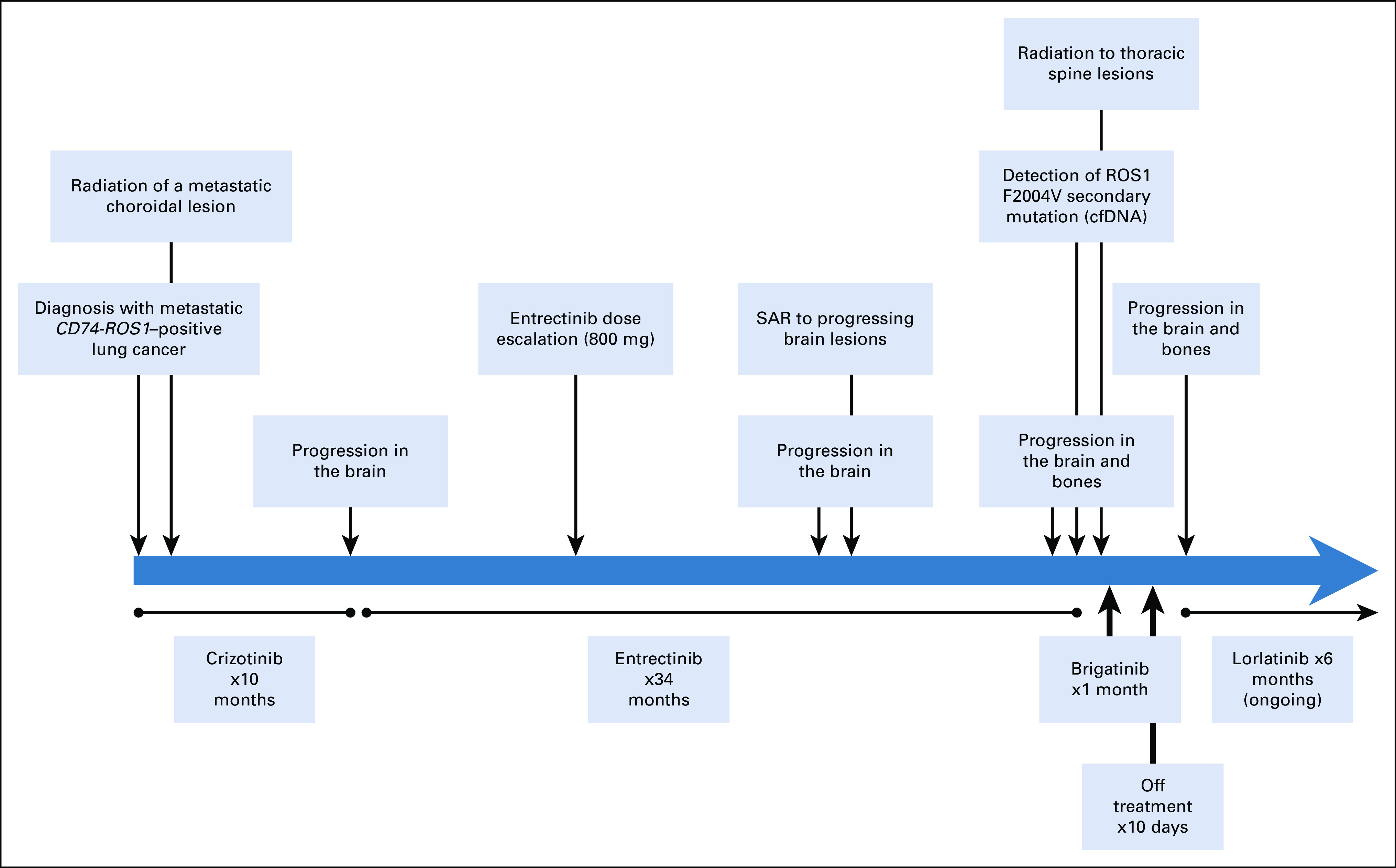
Timeline of the clinical course, highlighting the treatment interventions and the molecular data from the time of diagnosis. cfDNA, circulating cell-free DNA; SAR, stereotactic ablative radiation.

Although, to our knowledge, the *ROS1* F2004V has not been described before in patients, it is analogous to the *ALK* F1174L mutation described in patients with neuroblastoma^19^ and confers paradoxic apoptosis to Ba/F3 cells, a result of excessive oncogenic signaling through ROS1 and p38MAPK.^20^ Also, a secondary F1174V mutation has been described in ALK-positive lung cancer with resistance to crizotinib.^21^ Instead, Ba/F3 cells expressing ROS1 F2004V survive in the presence of low-dose ROS1 inhibitor.^20^ However, the patient in this report did not respond to withdrawal of brigatinib, and symptoms, namely headaches, developed from brain metastases. Disease burden in the brain was not amenable to stereotactic ablative radiation, and to avoid whole-brain radiation therapy, a decision was made to enroll the patient in a clinical trial (ClinicalTrials.gov identifier NCT03178071) of lorlatinib, another TKI with ROS1 activity.^22^ Lorlatinib induced a dramatic response in the brain and the systemic disease sites, evident in the first disease evaluation with imaging studies (Fig 1). Disease remains in control 6 months later, while the patient continues receiving lorlatinib (Fig 2).

## DISCUSSION

In the case presented, we describe a *ROS1* F2004V mutation in a patient with ROS1 fusion–positive lung adenocarcinoma after progression while receiving entrectinib. This mutation, along with F2075C, emerged as a possible mechanism of resistance to cabozantinib, a multikinase inhibitor with ROS1 activity in vitro.^20^ Although the patient in that study did not receive cabozantinib, it is likely that F2004V is a mechanism of secondary resistance to ROS1 inhibition with entrectinib. In that work, Ogura et al^20^ established Ba/F3 cells expressing *CD74-ROS1* and resistance to cabozantinib by exposure to *N*-ethyl-*N*-nitrosourea, a known mutagen. Interestingly, the resistant cell lines were TKI addicted, because they required the presence of low-dose cabozantinib to survive. Withdrawal of the TKI induced apoptosis, which was driven by excessive ROS1 signaling. Despite these experimental data, we did not observe clinical response in the patient upon withdrawal of brigatinib. Given that the concept of “TKI addiction” is suggested in preclinical models only,^23-25^ the approach of TKI withdrawal cannot be recommended unless there is additional clinical validation.

**FIG 3.**
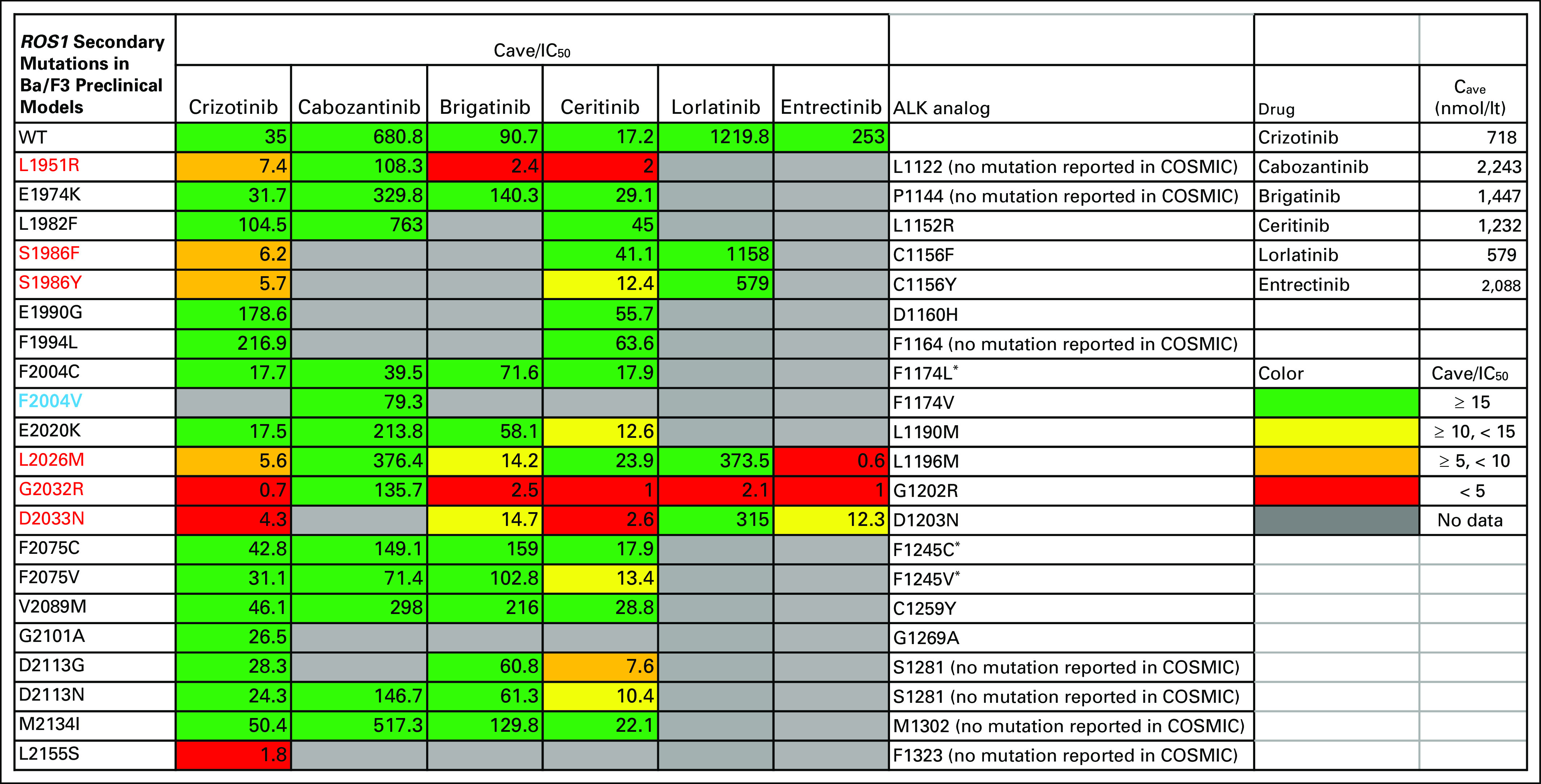
Secondary mutations in *ROS1* fusions reported in in vitro experiments in the literature and differential sensitivity to drugs with known ROS1 activity. The numbers in the boxes correspond to the average concentration (C_ave_) for the drug in steady state in patients, divided by the drug IC_50_ values calculated in Ba/F3 cells expressing the mutant ROS1 molecules. The half maximal inhibitory concentration (IC_50_) values were obtained from published reports. Colors represent relative potencies of the drugs compared with achievable drug exposure. The average IC_50_ was used if there was more than one publication for a certain mutation-drug combination. Repotrectinib is another potent ROS1 inhibitor with activity against many of these secondary mutations; it is not shown in the figure because the RP2D is not known yet for this drug.^29^ C_ave_ values were calculated as the ratio of the area under the curve for 0 to 24 hours to 24 hours of the drug at steady state in patients.^35,36^ Secondary mutations that have been reported in clinical cases are highlighted in red and the *ROS1* F2004V mutation described clinically for the first time in this report is highlighted in purple. The last column shows the homolog amino acid positions in ALK, estimated with the blastp program (https://blast.ncbi.nlm.nih.gov/Blast.cgi?PAGE=Proteins), and any *ALK* mutations at those positions reported in the literature or the Catalog of Somatic Mutations in Cancer (COSMIC) database. (*) These *ALK* mutations also are found in neuroblastoma.

The molecular spectrum of secondary resistance to crizotinib and other ROS1 inhibitors in ROS1 fusion–positive patients with lung cancer is not yet well established. The two largest published cohorts include 16 patients from Massachusetts General Hospital^26^ and 12 patients from the University of Colorado.^27^ In the Massachusetts General Hospital cohort, nine of 16 patients had a *ROS1* secondary mutation identified at the time of progression. Seven of these patients harbored the G2032R mutation and none had the F2004V mutation. Conversely, a *ROS1* compound secondary mutation (L2026M/L1951R) was identified in one of 12 patients in the University of Colorado cohort. In addition, in this cohort, there was also activation of the HER2 axis, an activating *KIT* D816G mutation and a *CTNNB1* S45F mutation, each case a possible mechanism of resistance to ROS1 inhibition. In three patients, there were copy-number gains in oncogenes, which might also represent mechanisms of resistance. Figure 3 summarizes the in vitro^10,16,20,28-33^ and clinical^10,27,30,34^ data of *ROS1* secondary mutations reported in the literature.

Molecular testing during the course of treatment with a targeted therapy often identifies molecular mechanisms of resistance to the inhibition of the driver oncogene.^1^ In the best-characterized cases of secondary resistance, there are data from large cohorts of patients and available effective TKIs, as is the case with *EGFR* T790M secondary mutation. Nevertheless, predicting response to a TKI in the presence of a secondary mutation in the driver oncogene might rely on less extensive clinical data or on preclinical data only. Patient-derived cell lines,^27^ Ba/F3 cells engineered to express and depend on the driver oncogene of interest with and without the secondary mutation,^10^ and xenograft tumors in mice^29^ serve as resistance models in many of these scenarios. In vitro development of resistance in cell-line models is possible by exposure to increasing doses of TKIs for several months.^37^ Alternatively, brief exposure to the mutagen *N*-ethyl-*N*-nitrosourea, followed by drug screening, can select for resistant clones.^20,28^ Finally, it is possible for a clinician to encounter a case of resistance for which molecular testing might identify resistance mechanisms not described before. Reports of such “n of 1” cases, including outcomes, is an important resource that can guide treatment of cases with rare acquired resistance mutations.

In conclusion, we show that *ROS1* F2004V secondary mutation confers resistance to entrectinib and brigatinib, and possibly other ROS1 inhibitors, but is sensitive to lorlatinib. This knowledge will help guide treatment of prospective patients with the same molecular background.
